# Nine year follow-up of a rare case of angioedema due to acquired C1-inhibitor deficiency with late onset and good response to attenuated androgen

**DOI:** 10.1186/s13223-018-0274-5

**Published:** 2018-10-25

**Authors:** Polliana Mihaela Leru, Vlad Florin Anton, Horia Bumbea

**Affiliations:** 1 Carol Davila University of Medicine and Pharmacy, Bulevardul Eroii Sanitari, no. 8, District 5, 050474 Bucharest, Romania; 2 Internal Medicine Department, Colentina Clinical Hospital, Sos. Stefan cel Mare, no. 19-21, District 2, 020125 Bucharest, Romania; 3 Emergency University Hospital, Splaiul Independentei, no. 169, District 5, 050098 Bucharest, Romania

**Keywords:** Acquired angioedema, Attenuated androgens, C1 inhibitor deficiency

## Abstract

**Background:**

Angioedema due to acquired deficiency of C1-inhibitor (C1-INH-AAE) is a rare disease sharing some clinical and laboratory similarities with hereditary angioedema, but with late onset and no positive family history. The underlining cause may be malignant or due to autoimmune diseases, but some cases remain idiopathic.

**Case presentation:**

We report a case of a 75 year old woman suffering from recurrent episodes of angioedema since the age of 66, considered first induced by treatment with angiotensin-converting-enzyme inhibitors (ACEI). She continued to have angioedema attacks during 6 years after discontinuation of ACEI, until evaluation in our clinic in 2014, when C1 inhibitor esterase (C1-INH) deficiency was confirmed. The extended medical evaluation for inflammatory, allergic, autoimmune and neoplasic diseases was negative. C1-INH and complement fraction C4 plasma levels were significantly decreased at all measurements, but no diagnostic criteria for diseases known to induce C1-INH deficiency could be found. We first initiated daily prophylactic treatment with tranexamic acid, with no amelioration after 3 months. During the last and most severe attack, with the first facial and laryngeal edema, we have switched to attenuated androgen danazol. The evolution was very good, with prompt remission of angioedema and significant increase of C1-INH and C4 plasma levels after 2 weeks of daily danazol use. She completed 3 years of continuous treatment with low daily maintenance dose of danazol (ongoing), with no angioedema attack. We closely monitored C1-INH and C4 plasma levels, possible danazol side effects and any signs suggesting late onset of C1-INH deficiency causal disease.

**Conclusion:**

We reported a particular case of rare angioedema due to acquired deficiency of C1-inhibitor, which has no clear cause after long follow-up, but good response to attenuated androgen. We concluded that the awareness of angioedema due to C1-INH deficiency should be increased within medical community and therapeutic options should be more clearly indicated and available for all diagnosed cases.

## Background

Angioedema not accompanied by urticaria is a distinct and potentially severe disease, which has many hereditary or acquired forms, raising difficult problems in medical practice. According to the recent classification of angioedema without urticaria, four types of acquired (AAE) and three types of hereditary angioedema (HAE) were identified as separate forms [1]. Based on the cause and mechanism, acquired angioedema without wheals may be: idiopathic histaminergic, idiopathic non-histaminergic, related to angiotensin-converting enzyme inhibitors and due to C1-INH deficiency [2]. The awareness of non-allergic (non-histamine-mediated) angioedema within medical community is very low, since most cases of angioedema accompanied or not by urticaria are generally considered allergies. Angioedema due to acquired deficiency of C1-INH (C1-INH-AAE) is a rare disease that may have some clinical and laboratory similarities with hereditary angioedema, but without family history and with onset after the age of 40 years. The entity was first described in 1972 by Caldwell, who reported two patients with acquired C1-INH deficiency associated with lymphosarcoma and paraproteinemia, one having a clinical picture similar to HAE [3]. Prevalence of angioedema due to acquired deficiency of C1-INH is lower than that of hereditary forms, being estimated at 1:10 of that of HAE, meaning around 1:500,000 [4]. In most of the cases, the acquired C1-INH deficiency is secondary to malignant tumors, usually lymphoma or to autoimmune disorders such as systemic lupus erythematosus. The pathophysiologic mechanisms are consumption of C1-INH and classical pathway complement components activation of contact system and bradikinin release, during attacks [5]. Autoantibodies neutralizing C1-INH function could be found in collagen vascular diseases [6]. In approximately 15% of cases, considered idiopathic, the cause of C1-INH deficiency remains unknown and angioedema may raise severe clinical and therapeutic problems [7]. The clinical picture consists in recurrent episodes of angioedema of the face, tongue and upper airways, although any part of the body can be involved [8]. Gastrointestinal swelling attacks are less common in C1-INH-AAE patients compared with HAE cases [9]. Laboratory tests confirming diagnosis are reduced C1-INH plasma levels and/or activity of C1-INH below 50%. Decreased plasma levels of complement fraction C4 and CH50 are regularly observed. Significant reduction of C4 plasma levels is almost invariably present during angioedema attacks. C1q is also frequently decreased in AAE, but is normal in HAE. The presence of cleaved C1-INH may give apparently normal C1-INH antigen in about 20% cases, making the diagnosis even more difficult [10].

## Case presentation

We report a case of a 75 year old woman addressed to Allergology Department of our hospital in January 2014 for recurrent episodes of angioedema since the age of 66, with progressively increased severity and frequency. It was first considered to be induced by treatment with angiotensin-converting-enzyme inhibitors (ACEI) for mild hypertension, but she continued to have angioedema attacks for the next 6 years after discontinuation of ACE, with progressive aggravation during the last year. The previous multiple evaluations by many specialists in other hospitals did not succeed to give a clear diagnosis and treatment.

**Fig. 1:**
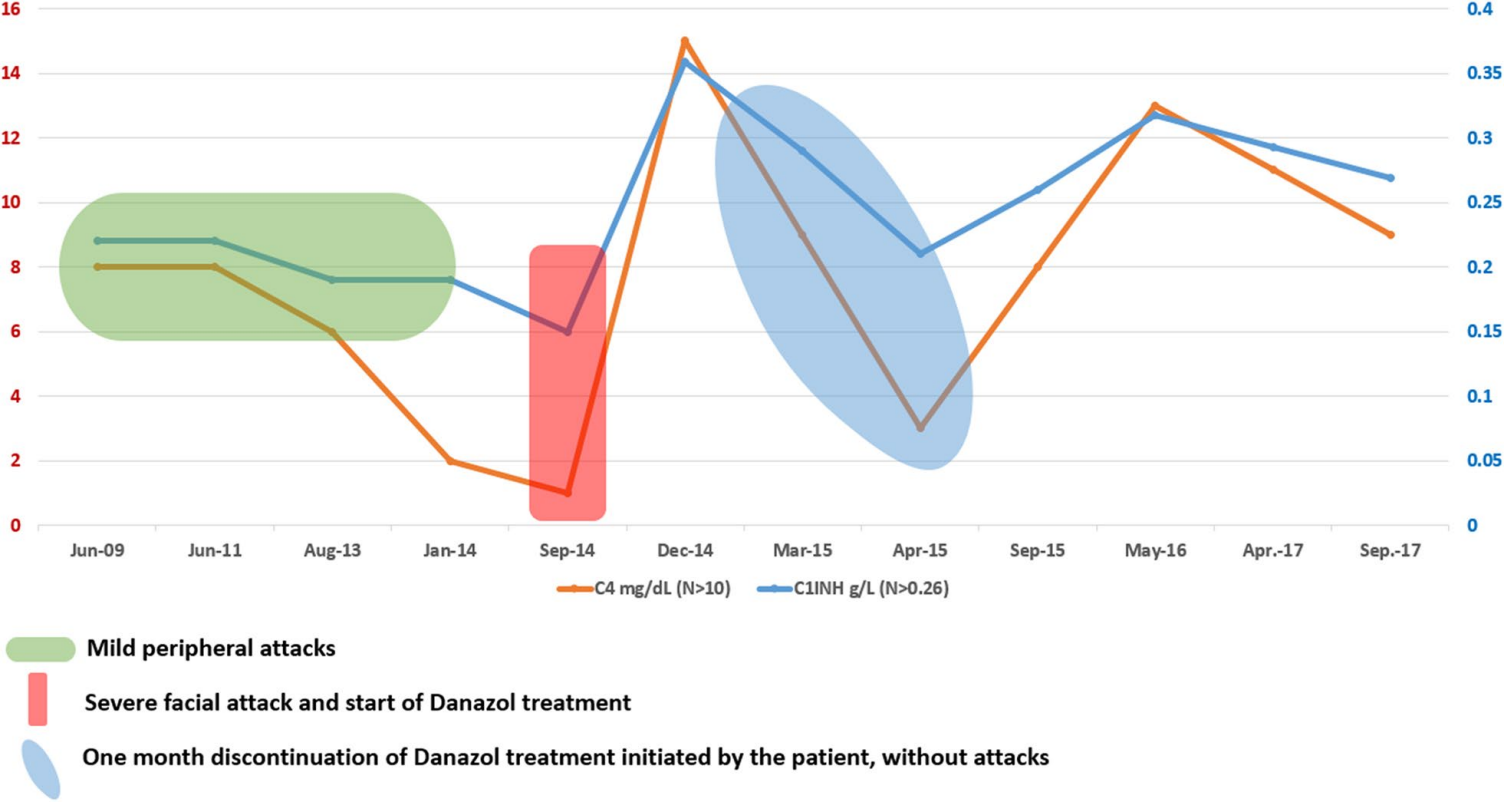
Serum C4 and C1-INH plasma level between 2009 and 2017

The patient had no relevant medical history and took no medication, except ACEI that was stopped some months after angioedema onset. No relation with possible allergic stimuli could be identified and she had no clinical manifestations between attacks. Angioedema was painful, not accompanied by urticaria or abdominal symptoms, located variably to neck, arms or buttocks, without facial involvement during 6 years. The attacks occurred at weeks or months intervals and usually lasted between 48 and 72 h, irrespective of corticosteroids and antihistamines treatment usually administered. The frequency of attacks had progressively increased from one at 2–3 months intervals to almost weekly during the last year before presentation. The last angioedema attack, determining emergency hospitalization in September 2014, was more severe and prolonged, accompanied for the first time by laryngeal edema and respiratory symptoms. The extended medical evaluation, including complete blood tests for inflammation, allergy, autoimmunity and cancer, were all negative (Table 1). Full body CT scan and bone marrow examination were normal. No criteria for lymphoproliferative, mieloproliferative or autoimmune diseases could be found. Measurement of C1 inhibitor (C1INH) in plasma showed significantly decreased level at all measurements, with low activity ranging from 58 to 4% and constantly low C4 (Fig. 1). Complement fraction C1q plasma level was measured twice and had normal value. Genetic tests were not performed, given the patient advanced age and lack of family history of angioedema, which are against HAE. A spontaneous mutation in SERPING 1 gene is noticed in up to 25% cases of HAE without family history, but we considered this probability very low in our case, due to late onset of angioedema.

**Table 1:** Laboratory tests for positive and differential diagnosis

Date	Laboratory tests	Other investigations
2008	ANA—negative Anticardiolipin antibody—normal Rheumatoid factor—normal	Colonoscopy—diverticulitis Mammography—normal Skin prick test—normal
2009	Serum diamineoxidase (DAO)—normal	Stop ACEI
2010	HBsAg, HCV-Ac—negative	Laparoscopic cholecystectomy
2011	CA 15.3, CA19.9, CEA—normal Papanicolau test—negative	
2012	C1q—normal Papanicolau test—negative	
2013	HBsAg, HCV-Ac—negative Serology for parasitic infectious—negative Total serum IgE and food specific IgE—normal DAO—normal CA 125—normal Anti-dsDNA—negative Electrophoresis and immunoelectrophoresis—normal Tryptase—normal	
2014	Complete blood count and biochemistry—normal CA15.3, CA19.9, CA 125, CEA—normal C1q—normal ANA—negative Anti-dsDNA—negative C3-normal Acute phase reactants—normal Rheumatoid factor—negative Thyroid profile—normal Lipid profile—normal LDH—normal	Full body CT—normal Bone marrow—normal

Treatment of angioedema attacks before hospitalization consisted of antihistamines and systemic corticosteroids, which proved to be ineffective. Since no pathogenic therapy with C1INH concentrate, antagonists of bradikinin receptors (icatibant) or selective inhibitor of plasma kallikrein (ecallantide) was available in 2014, we first initiated daily prophylactic treatment with tranexamic acid for 3 months, with no amelioration. During the more severe attack with laryngeal edema, in September 2014, we have switched to attenuated androgen danazol, given 400 mg the initial dose, reduced to 200 mg daily after 1 week and then to 100 mg daily. The clinical evolution was very good, no angioedema attack occurred since the introduction of danazol. C1INH and C4 plasma levels increased after 2 weeks of treatment and became normal after 1 month. After some months, the patient decided herself to discontinue danazol for short time, in order to check effects and to taper the minimal dose. Serum C1-INH and C4 plasma levels were measured after 2 and 4 weeks and showed significant lower levels, but no angioedema attack occurred during this period of time. She therefore restarted danazol prophylactic therapy at a minimum of 50 mg dose daily, ongoing after 3 years. The patient was closely monitored during the next 3 years, with complete clinical and laboratory control twice a year. We took into consideration the possible side effects of danazol, mainly dislipidemia, haematological and liver malignancies and any other complications or concomitant diseases. The clinical evaluation was very good, except two episodes of pulmonary cysts infection, remitted with broad spectrum antibiotherapy, which could not be related to danazol treatment. The clinical outcome of angioedema after 3 years of danazol treatment is very good, with no attacks or other related symptoms. No clinical or laboratory sign of any disease that could induce C1 INH deficiency occurred. No relevant side effects of danazol were noticed. The patient has an improved quality of life due to therapeutic compliance and general management plan.

## Discussion

Angioedema due to acquired C1-INH deficiency is a difficult diagnosis, generally established after many years of repeated attacks, with variable and potentially severe outcome, frequently accompanied or followed by lymphoproliferative disorders. The information about adult-onset non-allergic angioedema in medical practice is usually limited to ACEI-induced forms and the complex underlying mechanisms of the disease are poorly understood. The evidence that about 15% of patients with ACEI-induced angioedema may continue to have attacks during few months after ACEI discontinuation might be confusing and delay investigations for other causes of acquired angioedema. Data from the literature mention 58% patients with recurring attack within 3 months and 41% during the 1st month after ACE-I discontinuation, therefore alternate diagnosis might be considered if no significant clinical amelioration is seen after 1–3 months [11]. Before 1985 only 25 AAE cases have been published in the literature and the largest describing cohort of patients with AAE was reported 30 years later, consisting in 92 cases, with a median follow-up of 4.2 years after AAE diagnosis [12]. Acute attacks of AAE were primarily localized to the face and abdomen and half of the patients experienced potentially life-threatening attacks due to laryngeal or tongue localization. The authors reported a striking association of AAE with splenic marginal zone lymphoma, indolent B-cell lymphoma and monoclonal gammopathy. An important previous observation was that immunochemical underlying abnormalities and accompanying angioedema may precede with some years the clinical expression of malignant disease [13]. The main differences of AAE from HAE are advanced age, lack of family history, normal genetic tests, lack of triggers for attacks (such as trauma, infections and surgical or dental interventions) and more rare abdominal attacks [14]. Complement fraction C1q is usually low in AAE and normal in HAE, which is different from the findings in our case. We considered the utility of genetic test, but the cost is very high and it would not bring significant benefit to the case management, due to current available therapies for AAE.

Treatment of C1-INH-AAE should consider the underlying disease as well as the frequency and severity of angioedema. The current standard of care for AAE is based on experts recommendations [15]. Plasma-derived C1-INH, bradykinin B2 antagonist icatibant and kallikrein inhibitor ecallantide are recommended for treatment of AAE acute attacks, while antifibrinolytic agents such as tranexamic acid and attenuated androgens are indicated for long-term AAE prophylaxis.

Symptomatic treatment of C1-INH-AAE recurrences is similar to that of HAE, using C1-INH replacement therapy and bradykinin-targeted drugs, which may be not available or not approved for this indication. Long-term prophylaxis of AAE can be done with attenuated androgens or antifibrinolytic agents such as tranexamic acid, which is considered by some authors as the drug of choice for C1-INH-AAE [16]. Attenuated androgen danazol is a synthetic androgen used since 1976 in the long-term prophylaxis of HAE. It appears as a useful option for C1-INH- AAE also, due to the good safety and cost-efficacy profile [17]. Danazol was proved to increase the serum level of C1-INH in both HAE and AAE. The precise mechanism is not known, but it is considered to be mainly the decreased C1-INH consumption in AAE and increased liver synthesis in HAE [18].

A long-term survey of 118 HAE patients treated with danazol confirms a positive response in 94.1% patients, with good safety profile. The reported side effects are: weight gain, signs of virilization in women, altered lipid profile, hypertension, liver cell adenoma and slightly increased risk of stroke and myocardial infarction [19].The reported AAE case confirms the good safety profile of danazol treatment, which has proved to be an efficient and accessible therapeutic option for both AAE and HAE. No treatment for HAE and AAE was available in our country in 2014, except fresh frozen plasma for acute treatment, but tranexamic acid and danazol could be obtained from other European countries, based on medical prescription and for acceptable cost. For HAE treatment only, one C1-INH concentrate preparation was approved in 2015, followed by Icatibant in 2017.

## Conclusions

We reported a rare case of angioedema due to acquired C1-INH deficiency, with some particular aspects: 6 years diagnosis delay, initial peripheral localization of angioedema for many years, followed by one more severe facial and laryngeal attack, no abdominal attacks, no underlying disease after 9 years follow-up, lack of response to tranexamic acid and very good response to attenuated androgen.

The reported case is illustrative for various clinical presentations of recurrent angioedema in medical practice. The onset at advanced age and lack of any family or personal history of angioedema as well as negative allergologic investigation should prompt the physician to search underlying causes of C1-INH deficiency. The diagnosis, long term therapy and follow-up may be challenging and should take into consideration experts opinion and guidelines recommendations. We concluded that attenuated androgen is still an effective prophylactic choice, with good safety profile and low price. The main take-away message of our case report is that awareness of angioedema due to C1-INH deficiency should be increased within medical community and therapeutic options should be more clearly indicated and available for all diagnosed cases.
